# Efficacy and safety of HT048 and HT077 for body fat and weight loss in overweight adults: A study protocol for a double-blind, randomized, placebo-controlled trial

**DOI:** 10.1097/MD.0000000000017922

**Published:** 2019-11-11

**Authors:** Jungbin Song, Seon-mi Shin, Hocheol Kim

**Affiliations:** a Department of Herbal Pharmacology, Kyung Hee University College of Korean Medicine, Dongdaemun-gu, Seoul; b Department of Internal Medicine, Semyung University College of Korean Medicine, Jecheon-si, Chungcheongbuk-do, Republic of Korea.

**Keywords:** antiobesity, *Citrus unshiu*, clinical trial, *Crataegus pinnatifida*, *Nelumbo nucifera*, overweight, *Prunus persica*

## Abstract

**Background:**

The prevalence of excessive body weight has rapidly increased worldwide over the past decades; however, medications are intended for moderately and severely obese patients and are associated with side effects. As an alternative approach, the use of traditional herbal medicines has gained increasing popularity among overweight individuals in recent years in East Asia. HT048 is an herbal extract of *Citrus unshiu* and *Crataegus pinnatifida*, and HT077 is an herbal extract of *Nelumbo nucifera* and *Prunus persica*. These 4 herbs have been used widely for body weight reduction in China and Korea. The aims of this trial are to investigate whether HT048 and HT077 are effective at reducing body fat and weight in overweight adults, and to determine the safety of HT048 and HT077.

**Methods/design:**

A double-blind, randomized, placebo-controlled, 3-arm parallel group trial will be conducted in adults with a body mass index (BMI) of 25 to <30 kg/m^2^. A total of 120 eligible participants will be randomized in a 1:1:1 ratio to receive either HT048 (1000 mg), HT077 (400 mg), or matching placebo twice daily for 12 weeks, and will be monitored for an additional 4-week follow-up period after the treatment. All participants will be assessed for efficacy and safety of the investigational product at baseline and weeks 4, 8, 12, and 16. The primary endpoint is the change in body fat mass and percent body fat measured by dual-energy X-ray absorptiometry at week 12 from the baseline. The secondary efficacy variables are abdominal fat area measured by computed tomography, body fat mass and percent body fat measured by bioelectrical impedance analysis, body weight, BMI, and serum lipids and adipocytokines concentrations. Safety will be evaluated on the basis of reported adverse events, abnormal laboratory results, vital signs, and physical examination findings.

**Discussion:**

This is a first-in-human trial of HT048 and HT077 to assess the efficacy and safety in overweight subjects. The results will provide high-quality evidence of the therapeutic benefits of HT048 and HT077 for weight management and the prevention of obesity.

**Trial registration:**

Korean Clinical Research Information Service (KCT0004271) Registered September 2, 2019.

## 1 Introduction

The prevalence of overweight and obesity has reached epidemic levels in developed countries. In South Korea, overweight and obese adults defined as having a body mass index (BMI) ≥25 kg/m^2^ were one-third of the total adult population in 2015 and are predicted to reach approximately 50% by 2030.^[1]^ Excessive body weight predisposes to or is associated with various complications such as cardiovascular diseases, type 2 diabetes, hypertension, certain cancers, and osteoarthritis in weight-bearing joints.

Conventional weight management programs such as dietary modification and physical activity generally have low adherence and success rates and have been reported to often result in weight cycling.^[2]^ Medications for obesity include a lipase inhibitor (orlistat), appetite suppressants (eg, phentermine, lorcaserin), and a glucagon-like peptide-1 receptor agonist (liraglutide). These drugs are intended for moderately and severely obese patients with a BMI ≥30 or a BMI ≥27 with comorbidities and are associated with a high incidence of side effects.^[3]^ As alternatives to these medications, complementary and alternative treatments, such as herbs and acupuncture, have gained increasing popularity among overweight and obese individuals in recent years.^[4]^ In particular, traditional herbal medicines have been widely used in Asian countries, including Korea and China, for weight reduction.^[4]^

HT048 is an herbal extract of *Citrus unshiu* peel and *Crataegus pinnatifida* fruit, and HT077 is an herbal extract of *Nelumbo nucifera* leaf and *Prunus persica* flower. HT048 and HT077 have been developed by screening medicinal herbs used in East Asia for obesity treatment, with the purpose of reducing body fat and weight.

*C unshiu* peel and *C pinnatifida* fruit have been widely used for hundreds of years in traditional Chinese and Korean medicine, and have attracted increasing attention as promising agents for the treatment and prevention of obesity and metabolic diseases.^[5,6]^ The antiobesity and lipid-lowering effects of the 2 herbs have been well documented in experimental studies.^[6–8]^ Furthermore, *C unshiu* lowered BMI and improved lipid profiles in overweight and obese subjects,^[9]^ and a *C pinnatifida*-based herbal formula reduced plasma low-density lipoprotein cholesterol levels in dyslipidemic patients.^[10]^ Our previous animal studies showed more effective reduction in high-fat diet-induced body weight gain when *C unshiu* and *C pinnatifida* were combined compared to the individual herb.^[11]^ Their combination, HT048, reduced body weight, and adiposity to levels comparable to those in the orlistat, an antiobesity drug, -treated group and improved dyslipidemia in obese rats.^[11]^

*N nucifera* leaf has been traditionally used as folk medicine for the treatment of obesity, hyperlipidemia, and hyperglycemia in China,^[12,13]^ and *P persica* flower has been consumed as a tea for weight reduction in China and Korea.^[14]^ In preclinical studies, *N nucifera* has been demonstrated to exert antiobesity effects by inhibiting carbohydrate and lipid absorption, stimulating lipolysis via β-adrenergic activation, increasing thermogenesis, and downregulating lipogenic genes.^[12,13,15–17]^ We reported that *P persica* reduces adiposity and hyperglycemia in obese mice and is more effective when combined with *N nucifera*.^[14,18]^ HT077, the combination of these 2 herbs, exerted antiobesity effects and decreased elevated triglycerides and glucose levels in mice fed a high-fat diet.^[18]^

Although traditional use and preclinical data support the clinical application of HT048 and HT077, a prospective randomized trial has yet to be performed to investigate the efficacy and safety of HT048 and HT077.

### 1.1 Objectives

This study primarily aims to examine whether HT048 and HT077 are superior to the placebo in reducing body fat mass and percent body fat in overweight subjects. The secondary objectives are to assess the effects of HT048 and HT077 on abdominal fat, body weight, BMI, waist and hip circumference, and serum lipids and adipocytokines concentrations, and to investigate the safety of HT048 and HT077.

### 1.2 Design

This study is a double-blind, randomized, placebo-controlled, superiority trial with 3 parallel groups. Eligible participants will be randomized in a 1:1:1 ratio to receive either HT048, HT077, or placebo for 12 weeks, and will be monitored for an additional 4-week follow-up period after the treatment.

## 2 Methods and design

### 2.1 Study setting

The participants will be recruited from the Department of Internal Medicine, Semyung University Korean Medicine Hospital (Jecheon, Chungcheongbuk-do, Republic of Korea). The first patient was enrolled on September 6, 2019 and the recruitment period is expected to be 6 months from September. The recruitment announcement will be posted on the hospital homepage and bulletin board of the hospital to reach the target sample size.

### 2.2 Eligibility criteria

Participants who meet all the following criteria will be included.

- Men and women aged ≥19 and <60 years,
- BMI of 25 to <30 kg/m^2^,
- Participants who voluntarily decide to participate and sign the informed consent after being provided an explanation of this trial.

Participants who fall under any of the following conditions will be excluded.

- History of hypersensitivity to drugs or food ingredients,
- Participation in a commercial weight loss program or clinical trial for obesity within the last 3 months,
- Taking drugs or diet foods that affect their weight within 3 months before screening,
- Intentional weight loss of at least 5% within 3 months before screening,
- Undergone surgery, such as gastroplasty and intestinal resection, to lose weight within 6 months before screening,
- Endocrine diseases such as hypothyroidism and Cushing syndrome,
- Severe cerebrovascular disease (cerebral infarction, cerebral hemorrhage, etc), heart disease (angina pectoris, myocardial infarction, heart failure, arrhythmia in need of treatment), and lung disease (chronic obstructive pulmonary disease, etc) within the last 6 months. (However, those who are clinically stable may participate in the trial at the investigator's discretion),
- Serious dysfunction of the liver (alanine and aspartate aminotransferase levels of 2.5 times the upper limit of normal) or kidney (creatinine >2.0 mg/dL),
- Uncontrolled hypertension (blood pressure ≥160/100 mm Hg),
- Fasting blood glucose ≥126 mg/dL or random blood glucose ≥200 mg/dL, or diabetic patients taking oral hypoglycemic agents or insulin,
- Malignant tumor diagnosed within 3 years before screening,
- Complaint of severe gastrointestinal symptoms such as heartburn and indigestion,
- Psychologically significant medical history or current disease (schizophrenia, epilepsy, anorexia, bulimia, etc), or a history of alcohol or drug abuse,
- Determined to be unable to exercise because of musculoskeletal disorders,
- Pregnant or lactating women, or women of childbearing age who do not agree to use contraception during the trial,
- Participants who are deemed unable to comply with the test requirements or otherwise deemed unsuitable according to the investigator's opinion.

### 2.3 Procedures

**Figure 1:**
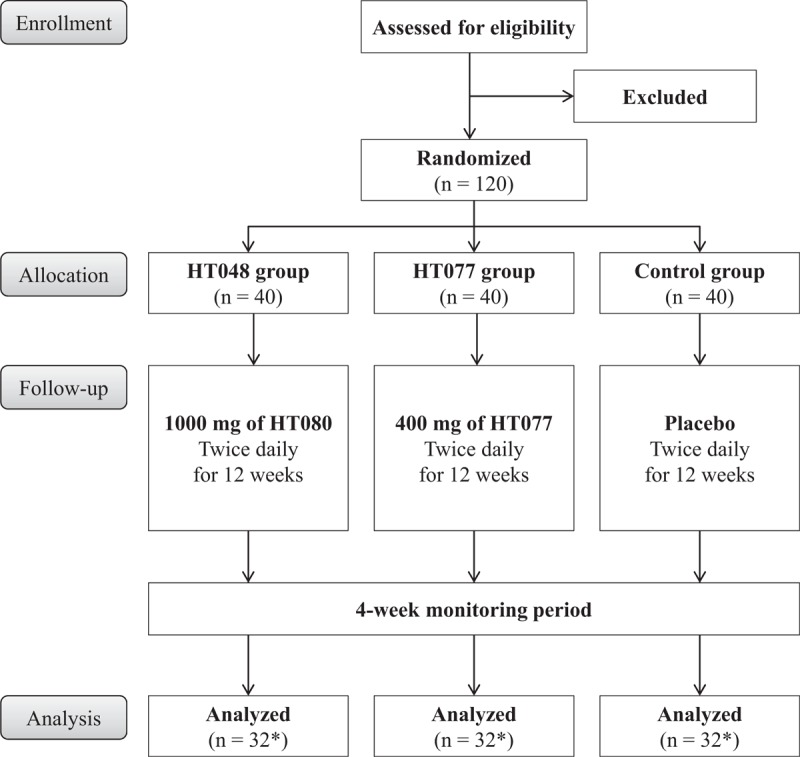
The study flow diagram. ^∗^The number of participants expected to complete the study.

This trial is designed to provide a 12-week treatment with either HT048, HT077, or placebo in overweight subjects and assess the reduction in body fat and weight for 16 weeks. The trial flow diagram is presented in Figure 1 and the schedule for enrollment, interventions, and assessment of this study is shown in Table 1. The principal investigator will obtain written informed consent from subjects who voluntarily agreed to participate in the study. Participants will be assessed for eligibility through screening tests at the first visit. Participants who meet the inclusion and exclusion criteria will undergo baseline assessments within 14 days after screening. At the baseline visit, participants will be randomly assigned to 1 of 3 groups: HT048, HT077, or placebo groups, with a 1:1:1 allocation ratio and will receive investigational products for 12 weeks. Participants will visit the hospital at weeks 4, 8, and 12 during treatment and will attend a follow-up visit 4 weeks after completing the treatment (at week 16). Efficacy and safety assessments will be conducted at baseline and weeks 4, 8, 12, and 16 as indicated in Table 1. All efficacy assessment items, including dual-energy X-ray absorptiometry (DXA), abdominal computed tomography (CT), bioelectrical impedance analysis (BIA), body weight, BMI, waist and hip circumference, and serum lipids and adipocytokines concentrations, will be performed on an empty stomach. Dietary intake and physical activity will be assessed using 24-hours dietary recall and international physical activity questionnaire short form, respectively, at baseline and weeks 12 and 16.

**Table 1:**
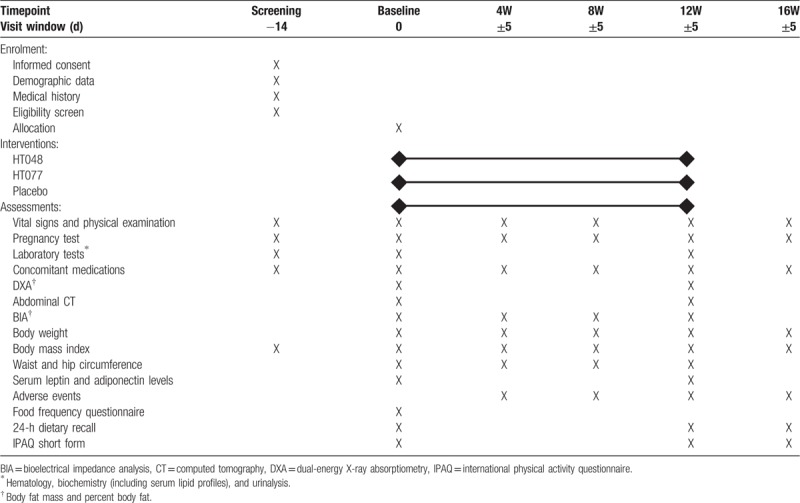
The schedule of enrollment, interventions, and assessments.

### 2.4 Interventions

The investigational product will be produced by COSMAX BIO Inc (Jecheon, Chungcheongbuk-do, Republic of Korea). The active product is a 500 mg tablet containing either 333.3 mg HT048 (125.8 mg of *C unshiu* extract and 207.5 mg of *C pinnatifida* extract) or 133.3 mg HT077 (57.3 mg of *N nucifera* extract and 76.0 mg of *P persica* extract). The active and placebo tablets are identical in shape, size, and color. Participants will be orally administered either 3 active or placebo tablets per dose, twice a day (morning and evening after meals) for 12 weeks. The daily doses are 2000 and 800 mg for HT048 and HT077, respectively. Participants will receive a 1-month supply of investigational product at baseline and weeks 4 and 8 and will be encouraged to continue to follow the prescribed dosage regimen. At weeks 4, 8, and 12, unused tablets will be returned and counted for the evaluation of participants’ compliance.

Participants will be advised to maintain their usual diet and exercise levels during the study. Participants will be prohibited to receive medicines and functional foods that can affect body weight and body fat during the study. Medicines, functional foods, exercise therapy, and diets that participants previously maintained before enrollment may be allowed if necessary at the investigator's discretion. Information regarding all concomitant medications, including the product or ingredient name, dosage, and duration, will be recorded at every visit.

Intervention will be discontinued under the following circumstances:

(1) if a serious adverse event occurs;
(2) if a participant has used a drug or physical procedure that could affect body weight and body fat;
(3) if a participant wish to discontinue study participation;
(4) if difficulties in assessments occur owing to administrative reasons, such as violations in dosage method or visit schedule; and
(5) if difficulties in follow-up occur owing to a participant's personal reasons.

### 2.5 Randomization and blinding

Participants determined to be eligible for this study will be randomly assigned to the HT048, HT077, or placebo groups in a 1:1:1 ratio using a computerized block randomization method. An independent statistician will generate the randomization sequence number and the block size will not be disclosed to the investigator for allocation concealment. Participants will be assigned a randomization number consecutively in the order in which they are enrolled and will receive the investigational product labeled with the same randomization number throughout the trial. The packaging and labels of investigational products for the 3 groups will remain the same for blinding purposes. Participants and all research personnel will be blinded to the assignment. The randomization sequence will be concealed in sealed opaque envelopes and will not be disclosed to the investigator until the end of the study, except when inevitable, such as when serious adverse events occur. If a code break occurs, the investigator will notify the contract research organization and the sponsor immediately and record the date and reason for the code-break in the case report form.

### 2.6 Outcome measures and endpoints

DXA will be used to assess body fat mass and percent body fat at baseline and week 12 as the primary outcome measure. In addition, body fat mass and percent body fat will be measured by BIA at baseline and weeks 4, 8, and 12. DXA and BIA are representative methods for assessing body composition, and the correlation between the 2 measurements for body fat mass and percent body fat are generally known to be good in both sexes.^[19,20]^ Abdominal CT will be used to measure visceral fat area, subcutaneous fat area, total abdominal fat area, and visceral fat/subcutaneous fat area ratio at baseline and week 12. CT scanning will be performed between the fourth and fifth lumbar vertebrae. With high accuracy, CT is considered as the reference method for measuring visceral and subcutaneous fat.^[21]^ Body weight and BMI will be measured at baseline and weeks 4, 8, 12, and 16. Waist circumference, hip circumference, and waist circumference/hip circumference ratio will be assessed at baseline and weeks 4, 8, and 12. According to the World Health Organization guidelines, waist circumference will be measured at the midpoint between the lower margin of the last palpable rib in the midaxillary line and the top of the iliac crest, and hip circumference will be measured at the largest circumference of the buttocks.^[22]^ Serum lipids (total cholesterol, triglyceride, high density lipoprotein cholesterol, and low-density lipoprotein cholesterol) and adipocytokines (leptin and adiponectin) concentrations will be assessed at baseline and week 12.

The primary efficacy endpoint is the change in body fat mass and percent body fat measured by DXA at week 12 from the baseline. The secondary efficacy endpoints are as follows:

(1) the change in body fat mass and percent body fat measured by BIA at weeks 4, 8, and 12 from the baseline;
(2) the change in visceral fat area, subcutaneous fat area, total abdominal fat area, and visceral fat/subcutaneous fat area ratio measured by abdominal CT at week 12 from the baseline;
(3) the change in body weight at weeks 4, 8, and 12 from the baseline;
(4) the change in BMI at weeks 4, 8, and 12 from the baseline;
(5) the change in waist circumference, hip circumference, and waist circumference/hip circumference ratio at weeks 4, 8, and 12 from the baseline;
(6) the change in blood lipids (total cholesterol, triglyceride, high density lipoprotein cholesterol, and low-density lipoprotein cholesterol) concentrations at week 12 from the baseline;
(7) the change in serum leptin and adiponectin concentrations at week 12 from the baseline;
(8) the change in body weight at week 16 from week 12; and
(9) the change in BMI at week 16 from week 12.

Safety will be assessed on the basis of adverse events reported by participants or the principal investigator, abnormal laboratory results (hematology, biochemistry, and urinalysis), vital signs, and physical examination findings. Laboratory tests will be performed at screening, baseline, and week 12. Measurements of vital signs and physical examination will be performed at every visit.

### 2.7 Sample size

This study aims to determine whether HT048 and HT077 is superior to placebo in reducing the change in percent body fat as measured by DXA at 12 weeks after the baseline measurement. The sample size has been estimated based on a previous study that evaluated the efficacy of an herbal extract powder for body fat reduction in overweight adults with a BMI of 25.0 to 29.9 kg/m^2^.^[23]^ The mean changes in percent body fat after the 12-week administration period are hypothesized to be 2.2% and 0.5% in the treatment and placebo groups, respectively, and the pooled standard deviation is hypothesized to be 2.14. Therefore, 25 participants in each group are needed to achieve a statistical power of 80% with a 2-sided significance level of 5%. To increase the power of the study, the sample size will be increased by 28%, with 32 participants in each group. Considering a drop-out rate of 20%, a total of 120 participants, 40 in each group, will be included in this study.

### 2.8 Statistical analysis

The efficacy outcome data will be analyzed by using both intention-to-treat and per-protocol population. The intention-to-treat population will comprise all randomized participants and the per-protocol population will be composed of participants whose primary efficacy assessments were performed according to the protocol without major protocol violations, such as unmet inclusion/exclusion criteria or the use of prohibited medication. Participants whose compliance to treatment is less than 80% will be excluded from the per-protocol population. The safety outcome data will be analyzed by using a safety population, which will include participants who receive treatment at least once and have at least 1 safety assessment after treatment. Missing data will be replaced using the last observation carried forward method.

Continuous variables will be described as mean, median, standard deviation, and range (minimum and maximum), and categorical variables will be described as frequency and percentage. Analysis of covariance with the baseline measurements as a covariate will be performed to examine inter-group differences in the primary and secondary endpoints. Continuous variables will be compared by using an independent *t* test or Wilcoxon rank-sum test between the treatment and placebo groups, depending on normality of the distribution. In addition, differences within groups before and after the intervention will be compared by using a paired *t* test or Wilcoxon signed-rank test. Categorical variables will be presented as the frequency and percentage and compared by using either the Chi-square or Fisher exact test. Adverse events will be classified by the Common Terminology Criteria for Adverse Events from the National Cancer Institute (ver. 4.0). Probability values of <.05 with 2-tailed tests will be considered significant. The statistical analysis will be performed with SPSS Statistics software version 25 (IBM, Chicago, IL). No interim analyses will be performed.

### 2.9 Data management and monitoring

The participating site will be regularly monitored by well-trained clinical research associates from a contract research organization in compliance with standard operating procedures. Source data verification during on-site monitoring visits, double data entry, and validation (eg, range checks) will be performed to ensure the data quality. All documents and data collected from participants will be kept confidential in a separate and secure location and will not be provided to any other third party without permission of the principal investigator and sponsor. A data monitoring committee will not be needed because the intervention is low risk and there will be no interim analyses.

### 2.10 Harms

All adverse events will be monitored and recorded, irrespective of causality. The principal investigator will evaluate the severity of symptoms and the causal relationship to the investigational product. If a serious adverse event occurs, it will be reported to the Institutional Review Board and sponsor within 24 hours, and the participation will be discontinued. Participants will be compensated for medical expenses related to injuries that are confirmed to be caused by the trial procedure or investigational products.

### 2.11 Withdrawal and dropout

Participants may withdraw at any time without penalty if they wish to discontinue their participation. The investigator may withdraw a participant if he/she considers it necessary for medical or administrative reasons (severe adverse events, significant protocol violations, etc). When a participant is withdrawn from the study, the date of withdrawal and the reason for withdrawal will be recorded.

### 2.12 Ethics and dissemination

This protocol (ver. 3.1, issue date August 13, 2019) has been approved by the Institutional Review Board of Semyung University Korean Medicine Hospital (approval no. SMJOH-2018-16-04) and registered at Korean Clinical Research Information Service (Identifier: KCT0004271). This trial will be conducted in compliance with the Declaration of Helsinki and the Korean Good Clinical Practice guidelines. Before proceeding with the trial, participants will be given detailed information about the trial and handwritten informed consent will be obtained from all the participants. The identity of participants will be protected by using a subject identification code in place of the participant's name in all documents. The principal investigator and sponsor will have access to the final trial dataset. The full trial protocol and datasets will not be publicly available but can be obtained from the corresponding author upon reasonable request. A model consent form will be also available on request from the corresponding author. The results of this study will be published in a peer-reviewed journal or presented at a conference.

## 3 Discussion

The prevalence of overweight and obesity has rapidly increased over the past decades worldwide and excessive body weight is a well-known risk factor for various diseases. Traditional herbal medicines are used widely in clinical practice for weight management in East Asia including Korea, but their use is contentious owing to the lack of high-quality evidence.^[4]^ Their efficacy and safety require well-designed and executed clinical trials. Here, we designed the protocol for a randomized controlled trial to investigate the efficacy and safety of HT048 and HT077 for the reduction of body fat and body weight in overweight Korean adults.

There are several limitations in this protocol. First, food intake and physical activity will not be controlled. However, the participants will be advised to maintain their usual patterns of eating and physical activity during this trial and self-report surveys on food intake and physical activity will be evaluated before and after administration. If there are any differences within and between the groups, adjusted analyses will be performed. Second, a short-term administration period has been selected, with a relatively small number of participants; thus, a larger, long-term trial is warranted to understand the impact on long-term weight and fat management.

Despite these limitations, one of the major strengths of this study lies in its prospective, double-blind, randomized, placebo-controlled design. Furthermore, BIA, DXA, and abdominal CT will be used to evaluate the efficacy of fat loss, thereby yielding more accurate and comprehensive results. BIA is simple, noninvasive, and inexpensive, but tends to underestimate body fat in overweight and obese subjects.^[20,24]^ Compared with BIA, DXA has greater accuracy and allows not only the quantification whole body fat, but also regional (ie, android, gynoid, trunk, arms, and legs) estimates of fat compartment.^[25]^ Visceral adiposity plays an important role in the pathogenesis of metabolic syndrome and is an independent predictor of mortality.^[26–28]^ CT allows visceral fat to be distinguished from subcutaneous fat with a greater precision than DXA and is considered the reference standard for the measurement of visceral adipose tissue.^[21]^ In addition, participants will be monitored for an additional 4-week follow-up period after the 12-week treatment to evaluate the maintenance of efficacy and safety. Some antiobesity mechanisms, such as appetite suppressants, can lead to withdrawal symptoms,^[29]^ but many herbal medicines lack follow-up studies after the end of administration.

This study is a first-in-human trial of HT048 and HT077 in overweight subjects. The results of this trial, which has a stringent design and high methodological quality, will provide evidence of the therapeutic benefits of HT048 and HT077 for weight management and the prevention of obesity.

## Acknowledgments

The authors would like to appreciate all the participants and research personnel for their support of this trial.

## Author contributions

**Conceptualization:** Hocheol Kim.

**Funding acquisition:** Seon-mi Shin.

**Methodology:** Jungbin Song, Seon-mi Shin.

**Project administration:** Seon-mi Shin.

**Supervision:** Seon-mi Shin.

**Writing – original draft:** Jungbin Song.

**Writing – review and editing:** Seon-mi Shin, Hocheol Kim.

Hocheol Kim orcid: 0000-0002-6690-0226.
